# Moyamoya disease presenting as thalamic infarction in a patient with neuromyelitis optica spectrum disorder

**DOI:** 10.1111/cns.13106

**Published:** 2019-02-06

**Authors:** Yin‐Xi Zhang, Yang Zheng, Meng‐Ting Cai, Lei Wu, Bao‐Rong Zhang

**Affiliations:** ^1^ Department of Neurology, Second Affiliated Hospital, School of Medicine Zhejiang University Hangzhou China

## 1 INTRODUCTION

Neuromyelitis optica is an immune‐mediated inflammatory demyelinating disease of the central nervous system typically involving optic nerves and spinal cord. With the discovery of the disease‐specific aquaporin‐4 (AQP4) antibody and the improvement of understanding of clinical and imaging patterns of brain involvement in what is now termed neuromyelitis optica spectrum disorder (NMOSD).^1^ The diagnosis of NMOSD is mainly based on the positive AQP4 antibody and the presence of at least one of the core clinical characteristics associated with optic nerve, spinal cord, area postrema, other brainstem, diencephalic, or cerebral presentations.^2^

Moyamoya disease (MMD), which was first reported by Japanese scholars Suzuki and Takaku in 1969, is a rare cerebrovascular disease characterized by bilateral progressive stenosis or occlusion of the terminal portion of the internal carotid artery and its main branches with emergence of coexisting abnormal net‐like vessels.^3^, ^4^ MMD is particularly common in Asian countries, especially in Japan. Cerebral ischemia and intracranial hemorrhage are two major hazards of the disease. The exact etiology and pathogenesis of MMD remain largely unknown.^5^ However, the coexistence with immunological diseases has been found among patients with MMD in recent reports.^6^ But there are few reports of patients with both diseases. Here, we describe the presence of MMD in a patient with NMOSD, suggesting autoimmunity may play a role in the occurrence and development of MMD.

## 2 CASE REPORT

**Figure 1:**
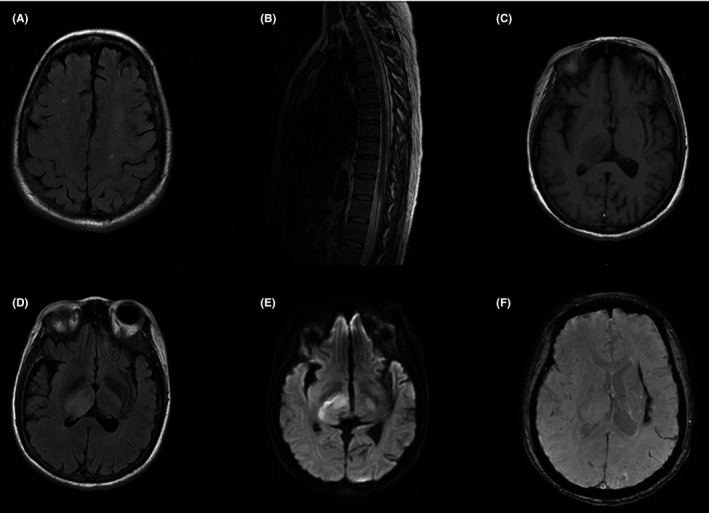
Magnetic resonance imaging (MRI) of the patient. (A, B) Previous MRI showed hyperintensive lesions in the centrum semiovale and thoracic spinal cord. (C‐E) MRI at this admission revealed right thalamic lesion, presenting with hypointensity on T1‐weighted images, hyperintensity on fluid‐attenuated inversion recovery sequences, and with restricted diffusion on diffusion‐weighted imaging. (F) Left basal ganglia hypointensity was observed on susceptibility weighted imaging, suggesting the deposition of hemosiderin after cerebral hemorrhage 3 years ago

**Figure 2:**
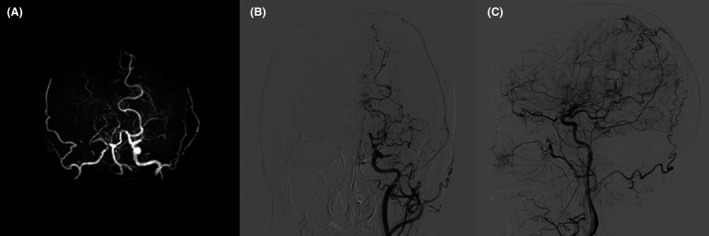
Cerebral vascular imaging. (A) Magnetic resonance angiography showed stenosis of the right intracranial segment of internal carotid artery and right posterior cerebral artery, with occlusion of the bilateral anterior cerebral artery and middle cerebral artery. (B, C) Digital subtraction angiography revealed stenosis of the left terminal internal carotid artery and “puff of smoke” angiogenesis

A 43‐year‐old female was admitted to our hospital complaining of numbness in the left limb for one week. Past medical history was notable for the diagnosis of NMO and intracranial hemorrhage. The patient was diagnosed with NMO ten years ago due to repeated episodes of blurred vision and numbness and weakness in the limbs. Brain and spinal magnetic resonance imaging (MRI) at that time indicated lesions in the brain white matter as well as spinal cord involving cervical and thoracic regions. AQP4 antibody test was not performed. Considering the potential diagnosis of demyelinating disease, steroid pulse therapy was initiated and the patient improved after the treatment. After discharge, corticosteroid was gradually tapered and the patient was maintained at a low‐dose corticosteroid and azathioprine in the long term. The patient was also diagnosed with left basal ganglia hemorrhage three years ago, presenting as right‐sided hemiplegia and confusion. Brain angiography was not performed at that time, and the patient recovered after symptomatic treatment. Family members exhibited no sign of the case pathology. Upon examination, the patient appeared lethargic and sluggish, with normal vital signs. Visual acuity was impaired in the right eye with a score of 20/200 on testing. Pupils were 3 mm bilaterally, round and reactive. Regarding motor function, muscle strength was decreased in the left extremities (Medical Research Council strength score, grade 3). Hypoesthesia of the left side was also observed. Babinski sign was present bilaterally. Further investigations revealed a positive AQP4 antibody in the serum, with an elevated titer of 1:32. An initial diagnosis of NMOSD was made considering her medical history and radiological findings. Other tests including complete blood count, basic metabolic panel, serum glucose, and anti‐nuclear antibodies were all normal. Brain MRI after admission indicated lesions with restricted diffusion in the right thalamus and hemosiderin deposition in the left basal ganglia (Figure 1). Unexpectedly, brain magnetic resonance angiography revealed severe stenosis of bilateral anterior and middle cerebral arteries, as well as stenosis of the right posterior artery and the intracranial segment of the right internal carotid artery. Radiological findings, as a result, strongly suggested the diagnosis of MMD. Digital subtraction angiography further confirmed this diagnosis with findings of bilateral occlusion of distal internal carotid arteries and rich collaterals near the skull base (Figure 2). After antiplatelet and other symptomatic therapies, the patient improved. She refused further treatment with vascular reconstruction surgery and received physical therapy at a local rehabilitation center.

## 3 DISCUSSION

The patient was diagnosed with brain infarction due to her clinical history (numbness and weakness of the left limb with an acute onset) and imaging findings (lesions with restricted diffusion on brain MRI). The underlying cause for the ischemic episode and the previous hemorrhagic event three years ago, as suggested by the angiographic findings this time, turned out to be MMD. Thorough investigations failed to yield any other cardiovascular risk factors, further corroborating the causative role of MMD. In addition, according to the 2015 diagnostic criteria,^2^ the patient also had concomitant NMOSD based on the recurrent episodes of blurred vision and limb weakness, radiographic lesions spanning the brain and spinal cord as well as a positive AQP4 antibody.

Neuromyelitis optica spectrum disorder is a humoral immunity‐mediated autoimmune disease, where AQP4 antibodies play a major role in‐between. AQP4 antibodies induce the infiltration of brain tissues by inflammatory cells through the activation of classic complement pathway, which further damages the astrocytes, oligodendrocytes, and neurons.^7^ By contrast, the pathophysiological mechanisms of MMD remain elusive. The development of MMD is a result of interplay between genetic, inflammatory, and autoimmune factors.^6^ Patients with MMD were reported to show positivity of several autoantibodies or concomitantly have other autoimmune diseases including Grave’s disease, systemic lupus erythematosus, Sjogren’s syndrome, antiphospholipid antibody syndrome, and type 1 diabetes.^5^, ^6^ Our patient with AQP4‐positive NMOSD further supports the role of autoimmune factors in MMD. The long‐standing NMOSD in this patient might accelerate the development of MMD and led to the ischemic event this time.

Neuromyelitis optica spectrum disorder and MMD rarely exist concomitantly in one patient. To our knowledge, this is the third report of patients with both diagnoses. The previous two patients with simultaneous NMOSD and MMD were both Asian females.^8^, ^9^ In fact, the two diseases share similar epidemiological features, with Asians and females more likely affected.^5^, ^10^ Genetic factors, therefore, may also explain the coexistence of the two diagnoses to some extent. However, the exact pathogenesis underlying the co‐occurrence of NMOSD and MMD remains unknown. More cases are needed to further elucidate this relationship in‐between.

## CONFLICT OF INTEREST

The authors declare no conflict of interest.
